# R-Baclofen Treatment Corrects Autistic-like Behavioral Deficits in the RjIbm(m):FH Fawn-Hooded Rat Strain

**DOI:** 10.3390/ph17070939

**Published:** 2024-07-13

**Authors:** Anita Varga, Rita Kedves, Katalin Sághy, Dénes Garab, Ferenc Zádor, Balázs Lendvai, György Lévay, Viktor Román

**Affiliations:** 1 Pharmacology and Drug Safety Research, Gedeon Richter Plc., Gyömrői út 19-21, 1103 Budapest, Hungary; 2 Doctoral School of Biology and Institute of Biology, Eötvös Loránd University, Pázmány Péter sétány 1/A, 1117 Budapest, Hungary; 3 Richter Department, Semmelweis University, Gyömrői út 19-21, 1103 Budapest, Hungary; 4 Department of Morphology and Physiology, Faculty of Health Sciences, Semmelweis University, Vas utca 17, 1088 Budapest, Hungary

**Keywords:** fawn-hooded rat, autism, asocial, hypersensitive, R-baclofen, disease model

## Abstract

The Fawn-hooded rat has long been used as a model for various peripheral and central disorders and the data available indicate that the social behavior of this strain may be compromised. However, a thorough description of the Fawn-hooded rat is unavailable in this regard. The objective of the present study was to investigate various aspects of the Fawn-hooded rat’s social behavior in depth. Our results show that several facets of socio-communicational behavior are impaired in the RjIbm(m):FH strain, including defective ultrasonic vocalizations in pups upon maternal deprivation, reduced social play in adolescence and impaired social novelty discrimination in adulthood. In addition, Fawn-hooded rats exhibited heightened tactile sensitivity and hyperactivity. The defects observed were comparable to those induced by prenatal valproate exposure, a widely utilized model of autism spectrum disorder. Further on, the pro-social drug R-baclofen (0.25–1 mg/kg) reversed the autistic-like defects observed in Fawn-hooded rats, specifically the deficiency in ultrasonic vocalization, tactile sensitivity and social novelty discrimination endpoints. In conclusion, the asocial, hypersensitive and hyperactive phenotype as well as the responsivity to R-baclofen indicate this variant of the Fawn-hooded rat strain may serve as a model of autism spectrum disorder and could be useful in the identification of novel drug candidates.

## 1. Introduction

The Fawn-hooded rat strain was derived from crosses between German brown, white Lashley and Long-Evans rats producing a phenotype that includes platelet storage pool deficiency, coagulation impairment, hypertension, proteinuria, renal failure and hypercortisolemia [1,2,3]. In addition to the physiological alterations, these rats have dysregulated serotonergic [4,5,6], dopaminergic [7], opioid [8], cannabinoid [9] and cholinergic [10] neurotransmission. There is also evidence that Fawn-hooded rats are characterized by central neuroinflammatory processes [11]. Behaviorally, Fawn-hooded rats may show a high preference for alcohol [12], increased immobility in the forced swim test, hyperactivity and reduced sucrose preference [6,11,13]. Based on this complex phenotype, the strain has been proposed as a model for renal and pulmonary hypertension [14], alcoholism [15], anxiety [16], depression [17] and mania [6]. Meanwhile, some of these findings have been challenged over the years [18,19]. It is now acknowledged that different substrains of Fawn-hooded rats may exhibit significant variation in their behavioral characteristics. For instance, while one substrain displays a depression-like phenotype, others may not [12,20].

In addition, Fawn-hooded rats have been described to display a socially avoidant behavior. Kantor and colleagues reported reduced social interaction compared to Sprague–Dawley rats, which is suggestive of a social phobia-like phenotype [21,22]. Social impairments are typical of a number of neuro-psychiatric conditions such as depression or alcoholism, for which the Fawn-hooded rat has been claimed as a preclinical model. Conversely, social defects are also hallmark features of autism spectrum disorder. While there are autism-inspired studies that have revealed a socially defective phenotype for several mouse strains [23,24], and some genetically modified rat lines [25,26,27,28,29], rat strains obtained by breeding have not been extensively investigated and reported in this respect [30]. Rats exhibit a more complex behavioral signature than mice [31,32] which warrants further investigation and the identification of autistic-like rat strains. On the other hand, valproate exposure early in the development can be considered as a risk factor of ASD [33], and the prenatal valproate treatment rat model has been utilized as a preclinical ASD model in several drug development programs aimed at treating core symptoms of this disease [34,35]. Given the paucity of information regarding the Fawn-hooded rat strain in this regard, the present study sought to investigate various aspects of its social and other autism-related behaviors.

In our studies we utilized the RjIbm(m):FH rat substrain derived from the FH/Wjd substrain from Janvier labs (Saint-Berthevin, France). This substrain was compared with control Wistar rats and rats exposed to prenatal valproate treatment, a widely accepted model of autism spectrum disorder [36,37]. With regards to social behavior, we investigated ultrasonic vocalizations of rat pups upon separation from the mother, adolescent social play and social novelty seeking in adulthood. Additionally, tactile sensitivity and locomotor activity were investigated to gain a more detailed understanding of the phenotype. To establish the predictive validity of the model, responsivity to the pro-social selective GABAB receptor agonist R-baclofen was investigated as a reference compound [38,39,40,41,42,43,44,45]. In summary, the rationale of the present study was twofold. Firstly, we sought to thoroughly characterize autism-related behavior in the Fawn-hooded rats. Secondly, we aimed to determine whether this strain could be used as an alternative to existing models for pharmaceutical screening purposes.

## 2. Results

### 2.1. Early Development

**Figure 1:**
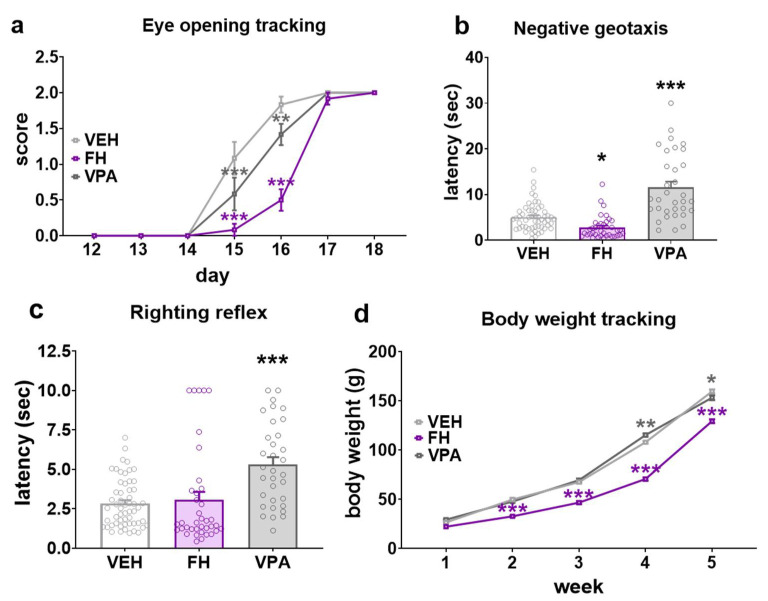
Early development in Fawn-hooded (FH) and prenatally valproate-treated rats (VPA) (*n* = 56 for vehicle control (VEH), *n*= 38 Fawn-hooded and *n* = 32 valproate-treated rats, respectively). Eye opening (**a**) was significantly prolonged in Fawn-hooded rats and somewhat accelerated in VPA-exposed pups during early postnatal development. Negative geotactic (**b**) and righting reflexes (**c**) had longer latencies in prenatally valproate exposed offspring. Conversely, latency of the negative geotactic reflex was shorter in Fawn-hooded rats compared to the vehicle control. The righting reflex of Fawn-hooded rats was unaltered. Fawn-hooded rats exhibited a reduced body weight gain (**d**) compared to both VPA-treated and control Wistar rats. *, **, *** *p* < 0.05, 0.01, 0.001 vs. Wistar controls (Dunnett’s multiple comparisons).

The general development of rats was followed by monitoring weight gain through postnatal week 1 to 5 and eye opening as a neurodevelopmental milestone during the period of PND12-18. Based on eye opening, Fawn-hooded rat pups exhibited a clear developmental delay (Figure 1a). The majority of the Wistar controls initiated eyelid opening on PND15 and had fully open eyes by PND16. A comparison of the developmental profiles of the Fawn-hooded and valproate-exposed Wistars with those of the controls revealed a shift towards an older age (Figure 1a; F(12, 231) = 10.03; *p* < 0.0001; two-way ANOVA). Prenatally valproate-exposed Wistars and Fawn-hooded pups exhibited fully open eyes at PND17 and PND18, respectively. Statistical analysis indicated a significant difference in terms of negative geotaxis (Figure 1b; F(2, 124) = 38.20; *p* < 0.0001; two-way ANOVA) and the righting reflex (Figure 1c; F(2, 124) = 12.20; *p* = 0.0001; two-way ANOVA). The righting and negative geotactic reflexes of Fawn-hooded rat pups were not found to differ from those of Wistar controls, indicating an intact motor development (*p* = 0.9581 and 0.2020, respectively; Dunnett’s test). Conversely, the latencies of righting and geotactic reflexes in prenatally valproate-exposed rat pups were significantly longer than those of the controls (*p* = 0.0002 and *p* < 0.0001, respectively; Dunnett’s test). With regards to body weight, Fawn-hooded rats also exhibited a delay in development as evidenced by a significant reduction in body weight gain in Fawn-hooded rat pups compared to Wistar controls on weeks 3, 4 and 5 (Figure 1d; F(8, 565) = 31.33; *p* < 0.0001; two-way ANOVA, *p* < 0.0001 each week; Dunnett’s test). Furthermore, the body weight gain of Fawn-hooded rat pups was also significantly lower compared to Wistars exposed to valproate prenatally on weeks 4 and 5 (*p* = 0.0075 and 0.0023, respectively; Dunnett’s test).

### 2.2. Ultrasonic Vocalization in Rat Pups

**Figure 2:**
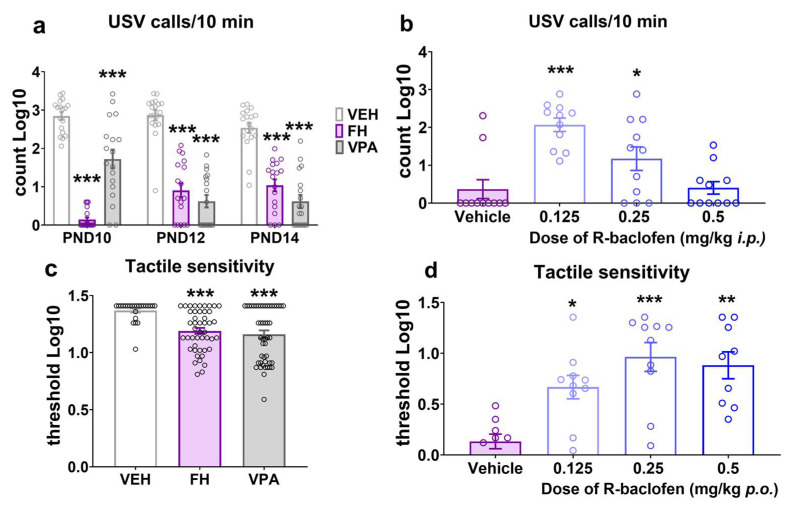
Both valproate-exposed and Fawn-hooded pups emitted fewer ultrasounds throughout early development when separated from their mothers (*n* = 18 for vehicle control, Fawn-hooded and valproate-treated rats, each) (**a**). Defective ultrasonic vocalizations could be partially reversed by 0.125 and 0.25 mg/kg ip. R-baclofen treatment in Fawn-hooded rat pups (*n* = 11) (**b**). Fawn-hooded and prenatally valproate-treated juvenile rats also showed reduced tactile sensitivity thresholds (*n* = 24 for vehicle control, *n* = 46 for Fawn-hooded and *n* = 48 for valproate-treated rats) (**c**). In Fawn-hooded rats, tactile hypersensitivity could be reduced by the administration of R-baclofen in the dose range of 0.125–0.5 mg/kg per os (*n* = 9–10) (**d**). *, **, *** *p* < 0.05, 0.01, 0.001 vs. Wistar controls (nonparametric Kruskal–Wallis test).

When separated from their mothers, rat pups emit ultrasounds, which represent a very early form of auditory communication that is aimed at eliciting maternal care. When the emission of ultrasounds is disrupted, it is indicative of socio-communicational and neurobehavioral abnormalities. In the present study both Fawn-hooded rat pups and Wistar rats prenatally exposed to valproate exhibited a robust reduction in ultrasonic vocalization compared to Wistar controls from PND10 up to PND14 (Figure 2a; F(4152) = 13.31; *p* < 0.0001; two-way ANOVA after log transformation of the data). While healthy Wistar rat pups typically exhibit a bell-shaped curve of ultrasonic vocalizations peaking around PND12, vocalizations of Fawn-hooded rat pups and Wistar pups with valproate syndrome are notably low throughout PND10 to PND14 (Figure 2a; PND10 *p* < 0.0001 and *p* < 0.0001, PND12 *p* < 0.0001 and *p* < 0.0001, PND14 *p* < 0.0001 and *p* < 0.0001, for Fawn-hooded and valproate rats, respectively; Dunnett’s test). The maternal deprivation-induced ultrasonic call counts were partially restored by R-baclofen to control levels in Fawn-hooded rat pups at the dose of 0.125 mg/kg and 0.25 mg/kg i.p. as compared to the vehicle (Figure 2b; F(3,40) = 11.86; *p* < 0.0001; two-way ANOVA after log transformation of the data). The highest dose tested (0.5 mg/kg) was not effective likely due to sedative side effects associated with this dose.

### 2.3. Tactile Sensitivity

Beyond the core symptoms of socio-communicational deficits and repetitive behaviors, ASD is often accompanied by sensory abnormalities including hypersensitivity to various modalities [33]. In this study, we used von Frey filaments on the skin of the hind paw to assess tactile sensitivity in juvenile rats (PND38-40). The results demonstrated a significant reduction in sensitivity thresholds in both the Fawn-hooded (Figure 2c; *p* = 0.0001 Kruskal–Wallis test after log transformation of the data) and the prenatally valproate-exposed Wistar rats (Figure 2c; *p* = 0.0001 Kruskal–Wallis test after log transformation of the data) compared to Wistar controls pointing at tactile hypersensitivity in these groups relative to the controls. R-baclofen demonstrated a near complete reversal of increased tactile sensitivity to control levels at the doses of 0.125, 0.25 and 0.5 mg/kg p.o. (Figure 2d; Kruskal–Wallis nonparametric test *p* = 0.0010, where 0.125 mg/kg *p* = 0.0457; 0.25 mg/kg *p* = 0.0007 and 0.5 mg/kg *p* = 0.0029 vs. vehicle; Kruskal–Wallis nonparametric test after log transformation of the data).

### 2.4. Spontaneous Motor Activity

**Figure 3:**
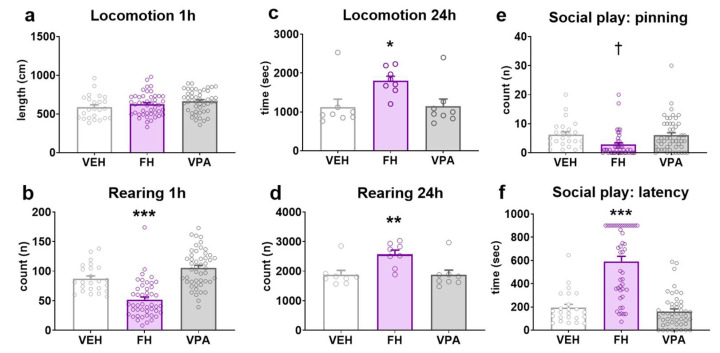
Spontaneous horizontal ambulation in an alien environment recorded for 1 h (*n* = 24 for vehicle control, *n*= 46 Fawn-hooded and *n* = 48 valproate-treated rats) (**a**) was not affected in either Fawn-hooded or prenatally valproate-exposed rats compared to Wistar controls. The number of rearing (**b**) during the same spontaneous locomotor assay was reduced in Fawn-hooded rats compared to controls, while valproate treatment did not affect this endpoint. In contrast to these, both locomotion (**c**) and rearing (**d**) were significantly increased in a home cage-like environment during a recording period of 24 h (*n* = 8 for each group). Social play behavior as measured by the number of pinning bouts (**e**) and latency to first playing bout (**f**) during a social interaction assay in adolescent rats was diminished in Fawn-hooded rats relative to controls. Prenatal exposure to valproate did not affect social play behavior. (*n* = 24 for vehicle control, *n* = 46 Fawn-hooded and *n* = 48 valproate-treated rats) *, **, *** *p* < 0.05, 0.01, 0.001 vs. Wistar controls (Dunnett’s multiple comparisons); † *p* < 0.05 vs. Wistar controls (non-parametric Mann–Whitney test).

In order to establish acute locomotor activity in a novel environment at a juvenile age, horizontal ambulation and vertical rearing behavior was investigated shortly after separation from the mother (PND26-28). The horizontal ambulatory behavior of the investigated groups was found to be similar during the 1 h recording period. Fawn-hooded or prenatally valproate-exposed Wistar rats did not differ significantly from control Wistars, indicating that both strains exhibited intact motor function in a novel environment (Figure 3a; F(2, 68) = 1.111; *p* = 0.3350; two-way ANOVA). Conversely, rearing responses were significantly diminished (by approximately 40–50%) in Fawn-hooded rats in comparison to the other two groups during the same recording session (Figure 3b; F(2, 68) = 44.03; *p* < 0.0001; two-way ANOVA, *p* < 0.0001; Dunnett’s test). This may be indicative of an anxious phenotype in response to a novel environment. Rearing activity in prenatally valproate-treated rats did not differ from that of controls (*p* = 0.0538; Dunnett’s test).

### 2.5. General Activity in the Home Cage

To investigate spontaneous locomotor activity in a home cage-like environment in adults, the behavior of rats was studied in a LABORAS^TM^ (Metris b.v. Hoofddorp, Netherlands) set-up at the age of PND49-52. To this end, the time engaged in locomotion and rearing behaviors was quantified. Fawn-hooded rats exhibited a hyperactive phenotype with respect to both locomotor activity (Figure 3c; F(2, 14) = 4.981; *p* = 0.0232; two-way ANOVA, *p* = 0.0275 vs. vehicle control; Dunnett’s test) and rearing behavior (Figure 3d; F(2, 14) = 8.271; *p* = 0.0043; two-way ANOVA, *p* = 0.0067 vs. vehicle control; Dunnett’s test).

### 2.6. Juvenile Social Play Behavior

Social play behavior represents the earliest form of social behavior that is oriented towards the peers and not the mother. It is a powerful indicator of adult social facilities [46]. Since the frequency of social play behavior shows a peak at adolescence, this behavior (number of pinning) was examined at the age of PND34-36. While social play behavior was comparable between Wistar rats prenatally exposed to valproate and their controls (Figure 3e; F(2, 68) = 5.173; *p* = 0.0081; two-way ANOVA, *p* = 0.8077; Mann–Whitney test), the number of pinning was significantly reduced in Fawn-hooded rats (*p* = 0.0195; Mann–Whitney test). Similarly, the latency to social play behavior was identical between Wistar controls and valproate-treated rats (Figure 3f; F(2, 68) = 51.35; *p* < 0.0001; two-way ANOVA, *p* = 0.8239; Dunnett’s test), Fawn-hooded rats commenced their first playing bout significantly later than controls (*p* < 0.0001; Dunnett’s test).

### 2.7. Sociability and Social Novelty Test

**Figure 4:**
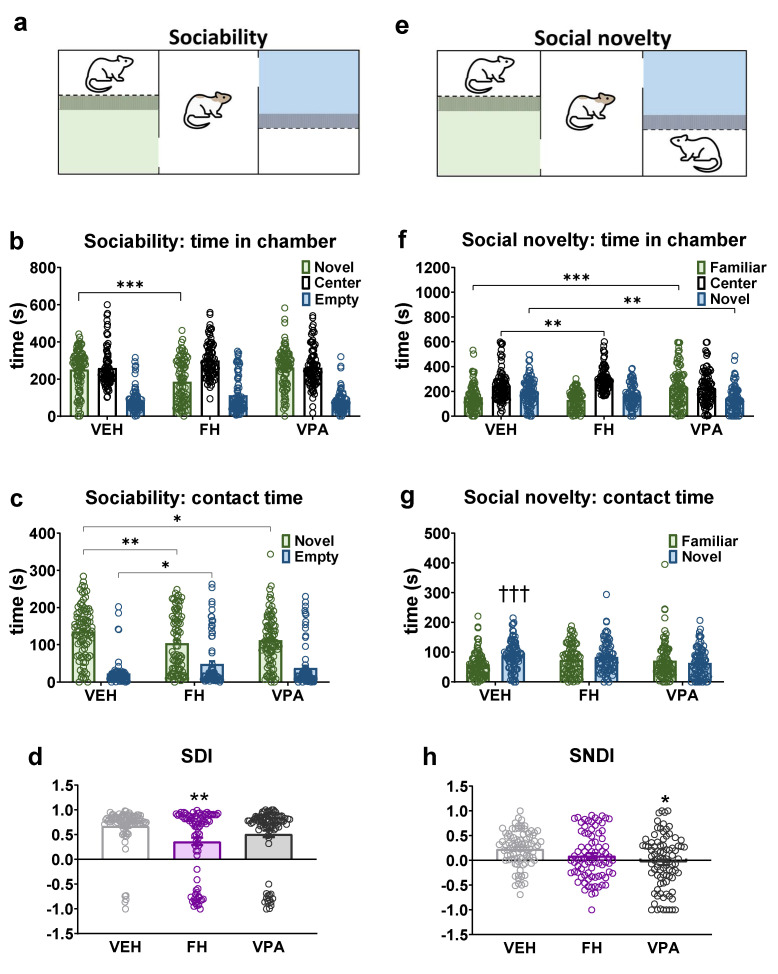
The sociability and social novelty seeking behaviors of Fawn-hooded and prenatally valproate-exposed Wistar rats were investigated in a three-chamber apparatus. The trial set-up for the sociability and social novelty phases is depicted in (**a**) and (**e**), respectively. The time spent in the social chamber during the sociability test phase was reduced in Fawn-hooded rats compared to controls (**b**). The time spent investigating a conspecific in the sociability phase was also diminished in both Fawn-hooded as well as valproate-exposed rats compared to controls (**c**). The sociability discrimination index (SDI) was significantly reduced in Fawn-hooded rats (**d**). Both Fawn-hooded and prenatally valproate-exposed rats demonstrated a reduced interest in a novel conspecific in terms of time spent in chambers (**f**) as well as time spent with direct investigation (**g**). The social novelty discrimination index (SNDI) experienced a non-significant reduction in Fawn-hooded rats and was significantly reduced in valproate rats compared to controls (**h**). *, **, *** *p* < 0.05, 0.01, 0.001 vs. Wistar controls (Dunnett’s multiple comparisons); ††† *p* < 0.001 vs. familiar (Šídák’s multiple comparisons test). Vehicle and VPA-treated group *n* = 88; Fawn-hooded group *n* = 83.

Rats are highly social animals and their capacity to detect and react to novel—as opposed to familiar—social stimuli provides an opportunity for adaptation in a rapidly changing environment. Inappropriate responses to novelty are associated with a number of neurodevelopmental and neuropsychiatric disorders including schizophrenia and autistic-related behaviors. Sociability and social novelty seeking were analyzed with the three-chamber apparatus (Figure 4a,e). The total distance traveled during each experimental phase remained consistent across all three groups.

In the sociability phase, Fawn-hooded rats spent significantly less time in the novel stimulus 1 lateral (Figure 4b; F(2, 768) = 257.8; *p* < 0.0001; two-way ANOVA, *p* < 0.0001; Dunnett’s test) and contact zone (Figure 4c; F(1, 512) = 209.5; *p* < 0.0001; two-way ANOVA, *p* = 0.003; Dunnett’s test) and spent significantly more time in the empty contact zone (Figure 4c; *p* = 0.0157; Dunnett’s test) than vehicle-treated Wistar rats. Wistar rats prenatally exposed to valproate also spent significantly less time in the novel stimulus 1 contact zone in the sociability phase, comparable to Fawn-hooded rats (Figure 4c; *p* = 0.028; Dunnett’s test). The diminished social interest exhibited by Fawn-hooded rats was also evidenced by the markedly reduced SDI parameter in comparison with vehicle-treated Wistar rats (Figure 4d; F(2, 252) = 5.676; *p* = 0.0039; one-way ANOVA, *p* = 0.0018 Dunnett’s test), a phenomenon that was observed in valproate-treated rats.

In the social novelty phase Fawn-hooded rats exhibited a significantly greater tendency to remain in the center zone compared to the vehicle-treated group (Figure 4f; F(2, 768) = 53.31; *p* < 0.0001; two-way ANOVA; *p* = 0.0013 Dunnett’s test), while Wistar rats with VPA syndrome spent a significantly greater amount of time in the familiar stimulus lateral zone (Figure 4f; *p* < 0.0001 Dunnett’s test) and significantly shorter duration in the novel stimulus 2 zone (Figure 4f; *p* = 0.0011 Dunnett’s test). In the contact zone, only the vehicle group demonstrated a significantly greater duration of contact with the novel stimulus animal 2 (Figure 4g; F(1, 512) = 6.099; *p* = 0.0139; two-way ANOVA, *p* = 0.0002; Šídák’s test). The SNDI was found to be reduced in Wistar rats prenatally exposed to valproate in comparison to the control group (Figure 4h; F(2, 249) = 3.041; *p* = 0.0496; one-way ANOVA, *p* = 0.0289; Dunnett’s test). Conversely, in the case of Fawn-hooded rats there was a tendency towards a reduction in this parameter, yet the alteration was not statistically significant in comparison to the vehicle (Figure 4h).

**Figure 5:**
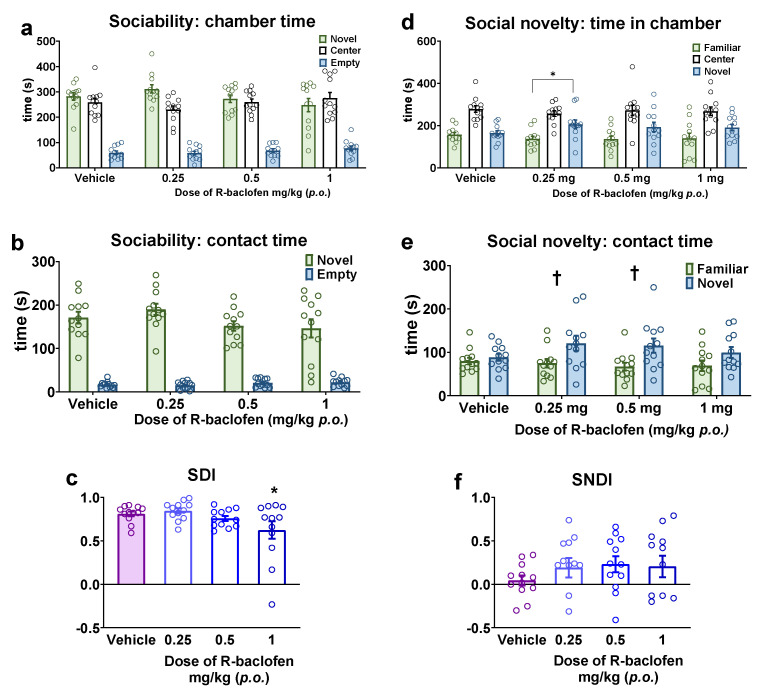
Sociability and social novelty seeking with Fawn-hooded rats following 0.25, 0.5 and 1 mg/kg R-baclofen administration compared to vehicle-treated group. Treatment with R-baclofen did not alter the cumulative time spent in the chambers of the three-chamber apparatus (**a**) or the time spent with direct investigation of a conspecific (**b**). The sociability discrimination index was reduced by the 1 mg/kg dose of R-baclofen (**c**). R-baclofen at the dose of 0.25 mg/kg increased the time spent in the chamber containing the novel stimulus rat (**d**). The 0.25 and 0.5 mg/kg doses of R-baclofen increased the time spent with direct investigation of the novel conspecific (**e**). R-baclofen treatment did not alter the social novelty discrimination index (SNDI) (**f**). * *p* < 0.05 vs. Wistar controls (Dunnett’s multiple comparisons); † *p* < 0.05 vs. familiar (Šídák’s multiple comparisons test). *n* = 12 for all treatment groups.

The subsequent step involved attempting to restore the reduced sociability observed in Fawn-hooded rats through the administration of R-baclofen treatment. However, the time spent in either zone did not show a significant difference when compared to the vehicle treatment (Figure 5a,b). On the other hand, the SDI was significantly reduced by R-baclofen at the 1 mg/kg dose in Fawn-hooded rats (Figure 5c; F(3, 44) = 3.598; *p* = 0.0207 one-way ANOVA; *p* = 0.0309, Dunnett’s test), which was not accompanied by reduced locomotor activity. Concurrently, in the social novelty phase, 0.25 mg/kg of R-baclofen significantly enhanced the time spent in the novel (stimulus 2) lateral chamber (Figure 5d; F(1, 88) = 14.89; *p* = 0.0002; two-way ANOVA, *p* = 0.0193; Šídák’s test) and contact zone (Figure 5e; F(1, 88) = 14.88; *p* = 0.0002; two-way ANOVA, *p* = 0.0375; Šídák’s test), revealing a pro-social character of the compound. Furthermore, the time spent in the novel stimulus 2 contact zone was also increased after treatment with 0.5 mg/kg R-baclofen (Figure 5e; *p* = 0.0259; Šídák’s test). The SNDI was not altered by R-baclofen (Figure 5f).

## 3. Discussion

The present study aimed to provide a comprehensive description of social and other autism-related behaviors in Fawn-hooded rats, as a thorough analysis of this strain had not yet been conducted. Indeed, Fawn-hooded rats exhibited a remarkably complex autistic-like phenotype on multiple behavioral endpoints across development comparable to that of the widely accepted prenatal valproate exposure model of ASD [37,47]. These results significantly contribute to the previously described social dysfunction in this strain [21,22]. In accordance with the diagnostic criteria and co-morbid conditions described in DSM-5 [33], the persistent socio-communicational problems, hyperactivity and tactile hypersensitivity provide sufficient face validity for the Fawn-hooded rat as a presumed model of ASD. Although the model appears to have an acceptable face validity at the symptomatic or behavioral level, this is limited, as no gene expression or other biochemical characterization was included in the present study.

Although inbred mouse strains were extensively studied earlier for their potential autistic-like behavior [23,24], in rat strains little work has been done. In particular, the Fawn-hooded strain has been left unexplored in this respect [30]. In the study by Ku et al. [33] Long Evans rats were observed to display reduced levels of playfulness in direct social interactions at juvenile and young adult ages in comparison to Sprague-Dawley rats, indicating that rat strains can indeed exhibit autistic-like phenotypes. Similar to the Long Evans rats, in our study, adolescent Fawn-hooded rats were observed to engage in significantly less play than Wistar controls and to exhibit equal levels of asociality as prenatally valproate-treated Wistar rats. The behavior of the Fawn-hooded rats is reminiscent of other, genetically based ASD models, including Shank2, FMRP or neuroligin-3 knockouts [26,48] or models induced by external factors such as prenatal valproate exposure or immune activation [47,49].

The three-chamber assay is a conventional method for assessing autistic-like behaviors in mice and rats [50,51]. In this assay, the Fawn-hooded rats exhibited a normal pattern of social preference comparable to that of the Wistar controls or the prenatally valproate-treated rats. However, the ratio of social preference was significantly less in the Fawn-hooded rats than in the two other groups. Conversely, the social discrimination behavior of Fawn-hooded rats, namely, the capacity or motivation to distinguish between a familiar and a novel conspecific was found to be impaired in comparison to Wistar controls. Social discrimination in the Fawn-hooded rat was as defective as in the rats prenatally exposed to valproate. In conclusion, the Fawn-hooded rats exhibited socially abnormal behavior in the three-chamber assay comparable to that observed in the valproate-treated Wistars. This behavior is comparable to that observed in other ASD models including MeCP2 overexpressing, MeCP2 duplication or Tsc2^+/−^ rats [29,52,53]. The Fawn-hooded rats in this study behaved remarkably parallel to MeCP2 duplication rats insomuch as their social preference for a conspecific versus and empty space was also relatively intact, while their ability to discriminate novel versus familiar was altogether lacking [53].

The pup-aged Fawn-hooded rats in the present study exhibited a clear developmental delay, as determined by eye opening. An early indication of abnormal development and autistic-like behavior was also observed in the form of defective ultrasonic vocalization upon separation from the dams, a phenomenon that has been described in a number of rodent ASD models [47,49]. Conversely, their geotactic and righting reflexes appeared to be intact, and their ambulatory behavior was comparable to that of the controls when they were juveniles. Interestingly, the rearing behavior of Fawn-hooded rats in a novel environment was reduced compared to the Wistar controls. The phenomenon of rearing behavior is complex in its causation, with numerous factors influencing its expression. These include curiosity, motor coordination, balance, altered internal states, malaise and the level of anxiety [54,55]. Although not within the scope of the present study, it is possible that anxiety may be a contributing factor to the reduced number of rearing observed. Previous studies have reported that the Fawn-hooded strain exhibits an increased level of anxiety, although contradictory results have also been documented [6,16]. It is therefore unclear whether the elevated anxiety observed in this strain may also have contributed to the social defects observed in the three-chamber test. This hypothesis is unlikely to be correct, as the overall social activity of the animals was maintained during the social preference phase of the three-chamber assay indicating that an increased level of general or social anxiety is not present in these Fawn-hooded rats. When the Fawn-hooded rats were observed in a home cage-like environment over a 24 h period at young adulthood, they exhibited increased ambulatory and rearing behavior, indicative of a hyperactive phenotype, which is often associated with ASD.

The Fawn-hooded rats in the present study also exhibited mechanical allodynia as evidenced by the von Frey filaments on the paw withdrawal thresholds. This feature may be important for translational reasons, as hypersensitivity on various modalities, including tactile sensitivity, can be an aspect of the autistic phenotype in humans [56]. Conversely, the Fawn-hooded rats are thereby similar to other autism-like preclinical models such as prenatal valproate exposure [47], cortex-specific deletion of Foxp1 [57] or other genetic modifications [58,59].

In addition to the symptomatic similarity between a preclinical model and the human disorder, a further important benchmark for a translationally sound model is its predictive validity, that is, whether the symptoms detected in the model respond to the administration of relevant drugs on the market. Unfortunately, such a gold standard compound is not currently available for the treatment of the core symptoms in autism spectrum disorder. Given the lack of an approved drug, we conducted an investigation with R-baclofen which has been shown to have clear effects in various preclinical models [41,42,43,44,45] and indications of efficacy in human studies [38,39,40]. Congruently with the earlier results in the public domain, R-baclofen in the present study reversed the early communicational deficit, restored tactile sensitivity and improved the social deficit in adulthood in the dose range of 0.125–0.5 mg/kg. The behavioral effects of R-baclofen are likely to be linked to its modulatory action on the excitatory-inhibitory (E-I) balance of the brain, which may be dysregulated in ASD [60]. While data on E-I processes are not available in Fawn-hooded rats, other rodent ASD models show defects in this respect [61]. R-baclofen, an orthosteric agonist of the GABAB receptors, may increase inhibition and thereby normalize the presumed E-I imbalance present in Fawn-hooded rats, similar to what was previously shown in humans [39,40]. Further studies are necessary to determine whether Fawn-hooded rats indeed present with an E-I imbalance and whether that can be modulated pharmacologically.

The improvement in socio-communicational and sensory behavioral endpoints by R-baclofen indicates a certain level of predictive validity for the Fawn-hooded rat. It is acknowledged that the results presented here are limited by the absence of a standard of care drug in the field and the use of a single reference compound. Nevertheless, these rats may be suitable for the testing of novel compounds in a pharmaceutical setting.

## 4. Materials and Methods

### 4.1. Experimental Animals and Husbandry

A breeding stock of consanguineous Fawn-hooded rats (FH/Wjd obtained from Janvier, France) was maintained at the local breeding colony of Gedeon Richter Plc. (Budapest, Hungary) Monogamous breeding pairs of a dam and a sire cohabited the cage with the litter. The litters were culled at 12 pups with the number of males maximized. Male offspring were separated after postnatal day (PND) 21. The Fawn-hooded rats used in the present study derived from the first to the twelfth generation.

Animals treated with VPA were maintained on a soy-free diet (Teklad soy protein-free rodent diet, ENVIGO, Madison, WI, USA) to preclude the potential neuroprotective effects of plant-derived estrogens. As with the Fawn-hooded rats, these litters were also culled at 12 pups with the number of males maximized. At PND21, the male offspring were separated.

All experimental animals in the present study were maintained on a 12–12 h dark–light cycle, with the light cycle commencing at 6 p.m. and an ambient temperature of 22–24 °C. The animals were provided with food and water ad libitum. All procedures were conducted by personnel who held the requisite licenses. The study protocol was approved by the Animal Research Ethics Committee of Gedeon Richter Plc. and Hungarian state authorities (PE/EA/648-7/2021). All efforts were made to comply with the 3Rs principle, which requires the reduction of the number of experimental animals involved as well as their discomfort and suffering. All experiments were conducted in strict compliance with the European Directive 2010/63/EU on the care and use of laboratory animals for experimental procedures.

### 4.2. Drug Administrations

R-baclofen (CPT-R4A34CBA, batch number 08042013, Capot Chemical Co., Ltd., Hangzhou, China) was stored at 4 °C. R-baclofen was administered intraperitoneally to rat pups (pretreatment time 30 min prior to USV measurements) and via oral gavage from 18 days of age (pretreatment time 1 h before von Frey and three-chamber measurements). R-baclofen was dissolved freshly on each experimental day before the start of the test sessions in saline or 2% Tween 80 suspended in distilled water. The doses and pretreatment time before testing were selected based on literature data and previous observations. For the vehicle controls, an equal volume of saline or 2%Tween 80 in distilled water was administered. The treatments and behavioral observations were conducted in a blinded manner.

To induce an autistic-like condition in the offspring, time-mated female Wistar rats (Janvier, France) were treated with VPA (Sigma, Dorset, UK, batch number: P4543-10G) intraperitoneally (i.p.) at a single dose of 300 mg/kg in a volume of 2.5 mL/kg physiological saline on gestational day 12.5. A saline-injected (i.p.) group was included as a vehicle control.

### 4.3. Developmental Observations

Gross developmental biological observations were conducted, including monitoring of body weight, eye opening, righting and negative geotactic reflexes. The body weight of rats was followed through weeks 1 to 5 postnatally. The status of eye opening was inspected daily from PND12 to PND18. The process of eye opening was followed by scoring, where two closed eyes received a score of 0, one open eye received a score of 1 and two open eyes received a score of 2. The righting and negative geotactic reflexes may be early indicators of motor (cerebellar and vestibular) development. Therefore, these reflexes were assessed from PND10 to PND14. To establish the righting reflex, rat pups were placed in a supine position on a plexiglass plane. The time required for the pups to transition from a supine to a prone position and to stand on four paws in contact with the plexiglass was recorded using a stopwatch. The cut-off time was determined to be 10 s. To assess negative geotaxis, each pup was placed on a rough slope (angle 20°) facing towards the bottom of the slope. The latency (seconds) taken by each pup to turn their body around by 180° was recorded using a stopwatch. The cut-off time was determined to be 30 s.

### 4.4. Maternal Deprivation-Induced Rat Pup USV

The ultrasonic calls of male rat pups (PND10-14, weighing 18–35 g) were measured in response to maternal deprivation. The litters with dams were placed in the experiment room one day prior to measurement. The animals were fed and allowed to drink ad libitum. The temperature of the experimental room was maintained at 22 °C. To induce the calls, the pups were separated from their mothers and placed individually into a normal rat cage for 10 min while calls were recorded by a bat microphone. Ultrasonic vocalization was audio filtered (low cut-off: 20,000 Hz, high cut-off: 50,000 Hz), recorded and quantified with Sonotrack software (version 2.4.0; Metris Bv, Hoofddorp, The Netherlands)

### 4.5. Spontaneous Locomotor Activity

Spontaneous locomotor activity was quantified at the age of PND26-28 in male rats [62]. The rats’ activity was monitored for 1 h using a six-channel activity monitor manufactured by Experimetria (Budapest, Hungary). The apparatus is comprised of acrylic cages (48.5 cm × 48.5 cm × 40 cm) outfitted with 2 × 30 pairs of photocells distributed along the entire bottom axis of the cage. In addition, arrays of photocells (30 pairs) are positioned at opposing sides of the cage at varying heights (6.5, 12, 18 and 23 cm) to detect rearing responses. The signals derived from photocell beam breakings are processed by a motion analysis software which determines the spatial position of the animal with 1 Hz sampling frequency and computes the time spent by the rats with ambulation and rearing counts for one hour at 15 min intervals. The animals were individually placed in one of the photocell cages for 1 h.

### 4.6. General Home Cage Activity

In this study, we employed the LABORAS^TM^ measuring instrument, a high-throughput system designed for the automated identification of multiple behavioral patterns. The system comprises a triangular-shaped sensor platform (690 × 690 × 976 × 13 mm), which is positioned on two orthogonally placed sensors and a third fixed point, attached to a bottom plate. The entire structure is supported by three poles, which are equipped with rubber feet to facilitate absorption of external vibrations. These feet are adjustable in height allowing for precise leveling. A type III cage (840 cm^2^) can be positioned on the sensor platform. The technology employes a methodology that measures the forces generated by the animal’s movements and subsequently converts these forces into behavioral classes and tracking information. The measurements were conducted using the LABORAS^TM^ 2.6.9 software with default settings. The animals were allowed to acclimate to the experimental room for a period of 24 h. They were then placed in the measuring cages between 8 a.m. and 11 a.m. and their behavior was monitored for a period of 24 h. Throughout the duration of the measurement, the rats had access to normal food and water.

### 4.7. Juvenile Social Play

The social play test was conducted at the age of PND34-36. The testing arena was a plexiglass cage (42 × 42 × 32 cm; l × w × h) with wood shavings covering the floor. The animals were paired with an unfamiliar partner, that is, a non-cage mate or non-litter mate. The body weights of the animals in a test pair differed by no more than 10 g. On PND34 and PND35 each animal was introduced to the arena for a period of 5 min individually. On PND36 the motivation for play was enhanced by isolating the animals for a period of 4 h prior to the commencement of the test. Animals that had been previously unfamiliar to each other were simultaneously placed into opposite corners of the arena. Their behavior was recorded for 15 min. The assessment of play behavior was conducted using the Observer 5.1 software (Noldus Information Technology B.V., Wageningen, The Netherlands). The most characteristic parameters of social play behavior, including the number of social play behavioral elements and the latency to the first bout of play behavior were scored [46].

### 4.8. Tactile Sensitivity

The measurement of the tactile withdrawal threshold was conducted using calibrated (force; g) von Frey monofilaments (Stoelting^TM^, Wood Dale, IL, USA) applied to the plantar surface of the left hind paw on PND38-40. Withdrawal threshold was determined by increasing and decreasing stimulus intensity and estimated using the Dixon up-down method [63,64]. The animals were placed in plexiglass cages with wire mesh bottoms to ensure access to the ventral surface of the hind paws. The von Frey filaments (0.4 g to 26 g) commencing with the 4 g filament, were gently pushed against the surface of the skin from below. The monofilaments were held at the position for a few seconds with enough force to cause a slight bend of the filament. A positive response in the animal model is indicated by a flinch of the leg. If the animal responds, the next filament in the series of decreasing stiffness is selected. If the animal does not respond, the next filament of increasing stiffness is selected. This process is repeated until a change in the pattern of responses is found, and five further responses are recorded following the same technique of selecting the next stiffer or less stiff filament dependent on the previous response. In the event that no change in response is observed (that is, responses are either all positive or negative) the cut-off value of 0.4 g for all positive responses or 26 g for all negative responses is applied. A 50% threshold is calculated from the obtained data using a custom-written macro in Excel (version 2308).

### 4.9. Three-Chamber Sociability and Social Novelty Test

The test is designed to assess sociability and social novelty seeking in the presence of a novel or familiar stimulus animal. The three-chamber apparatus was constructed from infrared-transparent black plexiglass and comprised three identical chambers with dimensions of 32.5/40/50 cm (length/width/height). The openings between compartments were sealed by guillotine doors. In the two side chambers, transparent and perforated plexiglass baffles were constructed to serve as enclosures for target animals (dimensions: 32.5/15/50 cm). The apparatus was placed on an infrared light source and the activity of the animals was video recorded from above and analyzed using Ethovision XT 11.5 software (Noldus Information Technology Bv., The Netherlands). Male Wistar rats of a similar age and lacking any prior contact with the subject rats were utilized as social stimulus animals. In the experimental protocol, subject rats were initially acclimated for a period of 5 min within the central chamber and then allowed to explore and acclimate to the entirety of the apparatus (habituation phase). The sociability phase (novel stimulus 1 vs. empty enclosure) and the social novelty phase (familiar stimulus 1 vs. novel stimulus 2) were each conducted for a period of 10 min. Following the conclusion of the habituation and the social preference test, the test animals were transitioned to the central zone. Between tests, the chambers were cleaned with paper, cotton wool and a 70% ethanol solution, followed by water. The time spent in each chamber, as well as the time spent exploring the targets (rat or empty enclosure), was analyzed. For the social preference test phase, a social discrimination index (SDI) was calculated as follows: SDI = (time spent in stranger contact zone − time spent in empty contact zone)/(time spent in stranger contact zone + time spent in empty contact zone). For the social novelty phase, a social novelty discrimination index (SNDI) was calculated as follows: SNDI = (time spent in novel contact zone − time spent in familiar contact zone)/(time spent in novel contact zone + time spent in familiar contact zone). Additionally, the cumulatively travelled distance was also monitored.

### 4.10. Statistical Analysis

The data on rat pup ultrasonic vocalization (USV) and tactile sensitivity were transformed logarithmically and analyzed using two-way ANOVA and Dunnett’s test for the USV data and Kruskal–Wallis test for the tactile sensitivity data. All early developmental endpoints, locomotor data and play latency were subjected to two-way ANOVA followed by post hoc Dunnett’s test. The number of play pinning instances was subjected to the non-parametric Mann-Whitney test. The statistical analysis of the three-chamber data involved the use of one- or two-way ANOVA followed by the post hoc Dunnett’s test or the Šidák’s test (for familiar vs. novel comparisons). The data are presented as mean ± standard error of the mean. Statistical significance was determined at *p* < 0.05. All data were analyzed using GraphPad Prism 10 (GraphPad software, Boston, MA, USA). No datapoints were excluded from the statistical analysis.

## 5. Conclusions

In conclusion, our findings suggest that the present substrain of the Fawn-hooded rat may serve as a behavioral model of ASD. While these animals did not exhibit an impaired phenotype on all behavioral endpoints, this does not preclude the substrain as an ASD model. Even genetically modified ASD models may demonstrate a complex phenotype, wherein not every aspect of behavior and physiology reflects autism. To illustrate, the neuroligin-3 knockout rat model exhibits a range of defects, yet its juvenile play behavior remains intact [65]. This underscores the importance of a comprehensive investigation of autism models, employing a multi-faceted approach to the autistic phenotype, which may enhance the prediction of a compound’s therapeutic efficacy in ASD patients. Moreover, our findings indicate that the Fawn-hooded rat is a more complex and robust model in comparison to prenatal VPA exposure. Furthermore, the Fawn-hooded rats demonstrated responsiveness to treatment with R-baclofen on multiple behaviors, indicating that this model may also be suitable for testing putative pharmacological treatments for ASD.

## Data Availability

The data sets generated or analyzed during the current study are available from the corresponding author upon reasonable request.
